# Enormes incidentalomes surrénaliens: rôle de l'imagerie médicale à propos de deux cas

**Published:** 2012-12-07

**Authors:** Zakari Nikièma, Aimé Arsène Yaméogo, Kouamé N'Goran, Rabiou Cissé

**Affiliations:** 1 Institut Supérieur des Sciences de la Santé / Université Polytechnique de Bobo-Dioulasso, Burkina Faso; 2 Service d'Imagerie Médicale, Centre Hospitalier Universitaire Souro Sanou, Bobo-Dioulasso, Burkina Faso; 3 Service de Cardiologie, Centre Hospitalier Universitaire Souro Sanou, Bobo-Dioulasso, Burkina Faso; 4 Faculté de Médecine, Université de Cocody, Abidjan, Cote d'Ivoire; 5 Service d'Imagerie Médicale, Centre Hospitalier Universitaire de Yopougon, Abidjan, Cote d'Ivoire; 6 Unite de Formation et de Recherche-Science De la Santé (UFR-SDS), Université de Ouagadougou, Burkina Faso; 7 Service d'Imagerie Médicale, Centre Hospitalier Universitaire Yalgado Ouédraogo, Ouagadougou, Burkina Faso

**Keywords:** Incidentalome surrénalien, Echographie, TDM, Difficultés diagnostiques, adrenal incidentalomas, Ultrasound, computed tomography, diagnostic difficulties

## Abstract

Les auteurs rapportent une série de deux cas d’énormes incidentalomes surrénaliens rarement observés dans la littérature. Ils étaient de siège bilatéral dans le premier cas et unilatéral droit dans le second. Si le diagnostic étiologique est parfois aisé dans les pays à haute technologie médicale, il pose un problème majeur dans certaines structures sanitaires des pays à ressources limitées. Le rôle essentiel de l'imagerie est d’établir de manière précise, peu invasive et peu coûteuse que possible, la nature bénigne ou maligne de l'incidentalome surrénalien. L’échographie et la tomodensitométrie ont permis de faire le diagnostic positif chez nos patients. La sémiologie tomodensitométrique est caractéristique quand elle associe la morpho-structure de la masse et deux critères majeurs basés d'une part sur l'histologie et la physiologie et d'autre part la cinétique de rehaussement de la masse après injection de produit de contraste. Toutefois, la biologie et surtout la biopsie assurent le diagnostic étiologique.

## Introduction

La terminologie incidentalome dérive étymologiquement de l'expression anglaise « incidental tumor ». Il s'agit d'une masse tumorale de découverte fortuite par imagerie médicale aussi appelée fortuitome. Les premières tumeurs en cause étaient des tumeurs surrénaliennes découvertes dans 4-10% des explorations d′imagerie [1]. Ensuite d'autres tumeurs comme les tumeurs hypophysaires, pancréatiques, thyroïdiennes, ovariennes, cardiaques et hépatiques ont été découvertes fortuitement. Finalement le concept a été élargi à toute découverte fortuite à l'occasion d'un examen réalisé en radiodiagnostic pour une autre affection [2–4]. La différenciation entre lésions bénignes et malignes est importante pour la prise en charge [5]. Ceci est d'autant important dans les pays à ressources limitées où le plateau technique est insuffisant. Cette situation est notée dans notre hôpital où il n'existe pas d'imagerie par résonance magnétique (IRM), d'imagerie nucléaire, de biologie de pointe et d'anatomopathologie. Nous décrivons deux cas d’énormes masses surrénaliennes rarement observées dans la littérature. Elles ont été explorées au CHU Souro-Sanou de Bobo-Dioulasso à l'aide d'un échographe standard et d'un scanner multicoupe de 6 barrettes avec un protocole en quatre phases dont la phase tardive était retardée de 10 minutes (mn). Nous discuterons des approches diagnostiques de l'imagerie au scanner.

## Patient et observation

### Observation 1

**Figure 1:**
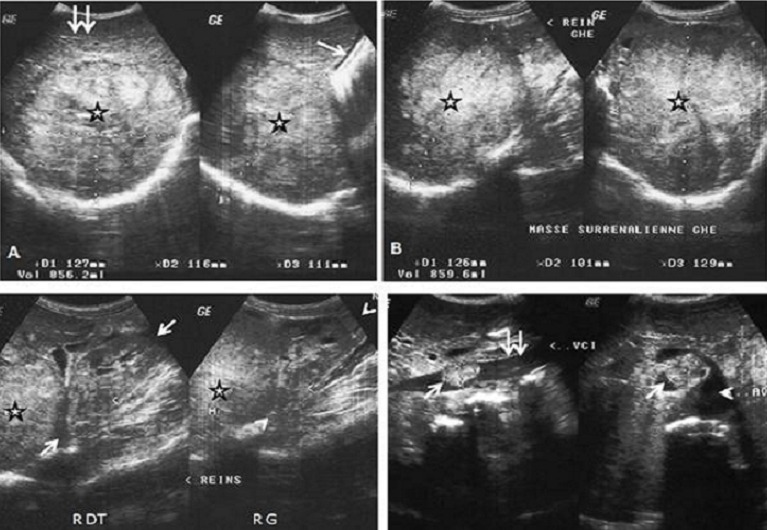
Observation 1. Echographie abdominale. Processus expansifs des loges surrénaliennes droite et gauche (étoiles, A, B, C), hyperéchogènes, hétérogènes et nécrosés. Ils mesurent 125 mm x 116 mm à droite et 126 mm x 101 mm à gauche. A noter un bourgeon tumoral de la veine cave inférieure sus rénale de 23 mm x 16 mm (flèches, D). A noter la vésicule biliaire (flèche, A), le foie (double flèches, A), le rein droit (flèches, C), le rein gauche (têtes de flèches, C) de conformation normale. Veine cave inférieure supra-rénale (double flèches, D). Aorte supra-rénale (tête de flèche, D). A: coupes transversale et longitudinale; B: coupes longitudinale et transversale; C: coupe longitudinale; D: coupes longitudinale et transversale

**Figure 2:**
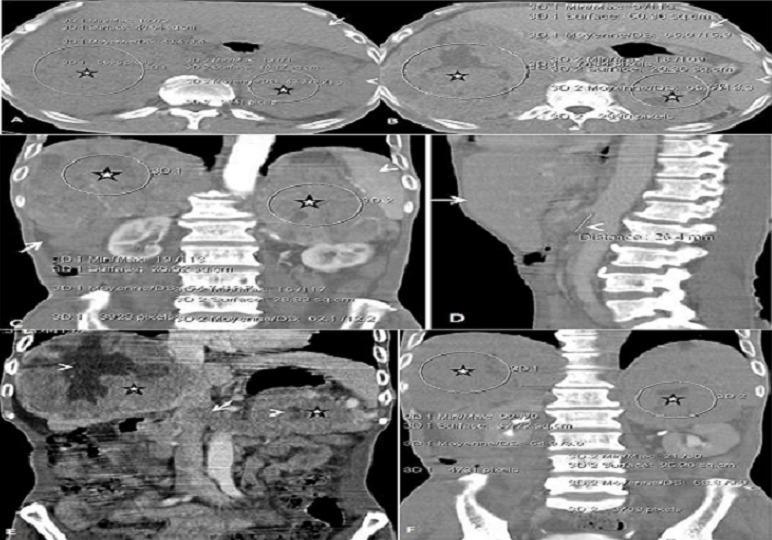
Observation 1. L'examen TDM abdominale réalisé sans et avec injection de produit de contraste montrait des volumineux processus expansif des loges surrénaliennes à structure dense de 43 UH avec une nécrose centrale autant à droite qu’à gauche (têtes de flèches, E) sur la séquence sans injection (étoiles, A). Rehaussement après injection de produit de contraste à la phase artérielle de 57 UH à droite (étoile, C), et de 63 UH à gauche (étoile, C), qui persiste à la phase portale de 65 UH des deux cotés (étoiles, B), et à la phase tardive à 10 mn après injection de 51 UH (étoile, F), et 61 UH (étoile, F), respectivement. Le wash out absolu est de 27% et le relatif de 9% faisant évoquer une tumeur maligne. A noter un bourgeon tumoral de la veine cave inférieure supra- rénale (flèches, F)., une adénomégalie cœlio-mésentérique (tête de flèche, D) et une lame d'ascite (flèches, C). Le foie (flèche, A, B, D) et le rate sont normaux (tête de flèche, A, B, C). A: coupe axiale sans injection de produit de contraste; B: coupe axiale avec injection à la phase artérielle; C: coupe coronale avec injection à la phase artérielle; D: coupe sagittale avec injection à la phase portale; E: coupe coronale avec injection à la phase portale; F: coupe coronale avec injection à la phase tardive

Homme de 65 ans, sans antécédents particuliers a été référé en cardiologie pour syndrome oedémato-ascitique. Le début de la maladie remonterait à plus d'un an. Elle se caractérisait par une anorexie, une asthénie, des douleurs abdominales dans un contexte de perte pondérale importante non chiffrée. L'examen clinique notait une hypertension artérielle labile, une énorme masse tumorale des deux hypochondres. Le bilan biologique usuel était non contributif. Les dosages des catécholamines urinaires de 24 heures, des dérivés méthoxylés et normétanéphrine n'ont pu être effectués. Une échographie abdominale (Figure 1) révélait un foie, une rate et des reins de conformation normale, refoulés par des volumineux processus des loges surrénaliennes hétérogènes qui étaient assortis de foyers nécrotiques. Ils étaient irréguliers et mesuraient 125 mm x 116 mm à droite et 126 mm x 101 mm à gauche. Il était à noter un bourgeon tumoral de la veine cave inférieure sus rénale de 23mm x 16mm et des adénomégalies retropéritonéales (Figure 1). L'imagerie échographique était suspecte à priori d'un corticosurrénalome malin avec une atteinte métastatique au niveau de la veine cave inférieure sus-rénale et ganglionnaire retropéritonéale. Toutefois, un diagnostic différentiel se pose avec les volumineuses masses surrénaliennes telles le schwannome géant, le liposarcome, le fibrosarcome, le lymphangiome ou le paragangliome. L'examen à la tomodensitométrie (TDM) réalisée sans et avec injection de produit de contraste en quatre phases (Figure 2) a permis d'affirmer l'origine surrénalienne et la caractérisation de la masse. Il s'agit d'une masse surrénalienne bilatérale de nature tissulaire nécrotique avec les mêmes aspects qu’à l’échographie.

### Observation 2

**Figure 3:**
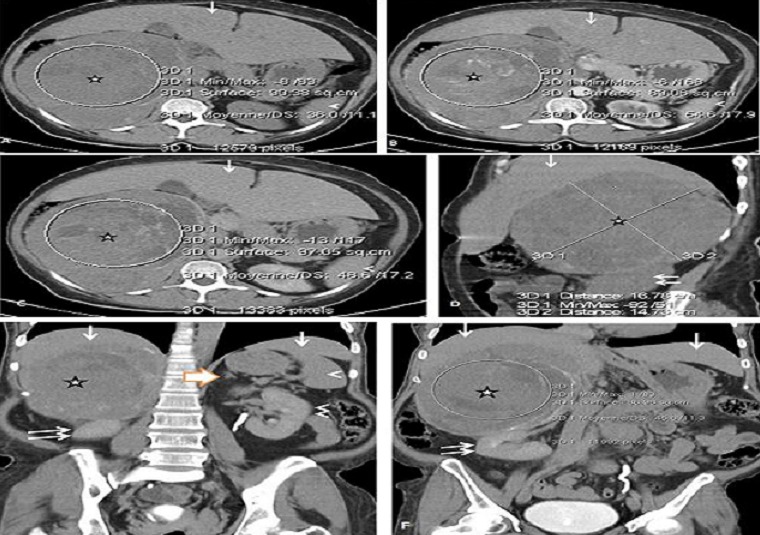
Observation 2. Mise en évidence d'une volumineuse masse surrénalienne droite de 167 mm x147 mm, régulière, tissulaire de 36 UH, hétérogène, nécrosée, sillonnée de calcifications (étoile, A, B, C, E, F), avec un rehaussement hétérogène à 54 UH sur la séquence artérielle (étoile, B), qui persiste à la phase portale de 48 UH (étoile, C), et de 46 à la phase tardive à10 mn (étoile, E, F). Le wash out absolu est de 16% et le relatif de 4% faisant évoquer un carcinome surrénalien. A noter une hépatomégalie homogène (flèche, A, B, D, E, F), une surrénale gauche normale (grosse flèche, E), une rate (tête de flèche, A, B, C, E), un rein droit (double flèches, D) et un rein gauche (double têtes de flèches, D) de conformation normale. A: coupe axiale sans injection de produit de contraste; B: coupe axiale avec injection à la phase artérielle; C: coupe axiale avec injection à la phase portale; D: coupe sagittale sans injection de produit de contraste; E: coupe coronale avec injection à la phase tardive; F: coupe coronale avec injection à la phase tardive.

Femme de 43 ans, sans antécédents particuliers qui consultait pour la première fois en cardiologie. Le début de la maladie remonterait à plus de deux mois par des douleurs de l'hypochondre droit. L'examen clinique révélait un état général conservé, une tension artérielle normale, une énorme hépatomégalie ferme sur syndrome d'insuffisance cardiaque droite qui a été documentée. Le bilan biologique usuel était non contributif. Les dosages des catécholamines urinaires de 24 heures, des dérivés méthoxylés et normétanéphrine n'ont pu être effectués. A l’étude échographique, on notait une énorme tumeur surrénalienne droite évaluée à 1200cc qui était irrégulière, tissulaire avec des foyers nécrotique et sillonnée de lisérés de calcifications. Le foie était de conformation normale. La TDM réalisée sans et avec injection de produit de contraste en quatre phases notait les mêmes aspects qu’à l’échographie (Figure 3).

## Discussion

Dans le cadre d'une masse surrénalienne de découverte fortuite la caractérisation de la lésion sera l'enjeu principal. Par contre, si le patient vient pour mise au point de troubles hormonaux, c'est la détection d'une lésion qui primera dans la mise au point.

Les moyens d'imagerie médicale constituent des outils d'exploration d'une importance majeure dans la découverte et l'approche diagnostique de ces masses surrénaliennes. Parmi ces modalités, la TDM et l'IRM ont pour avantages de mieux caractériser la masse, de préciser ses rapports avec les organes de voisinage, d'analyser les régions difficiles à explorer à l’échographie par les reconstructions multiplanaires et d’établir un bilan oncologique. Aussi, le rôle essentiel de l'imagerie est d’établir de manière précise, peu invasive et peu coûteuse possible, la nature bénigne ou maligne d'un incidentalome surrénalien [6, 7].

Ces tumeurs surrénaliennes sont souvent asymptomatiques et définies comme des «incidentalomes» avec une prévalence estimée à 4-10% en TDM [1]. L’échographie abdominale de part ses caractères intrinsèques est la technique utilisée pour rechercher et décrire des masses surrénaliennes de plus 20 mm. Toutefois, elle peut ignorer la masse surrénalienne [6]. Parmi les techniques de mesure de la densité spontanée, diverses études ont conclu que l′imagerie TDM est la procédure de choix pour l′évaluation de la glande surrénale [6]. Mais l′IRM qui possède certains avantages a été proposée récemment comme l'examen de choix car elle facilite la distinction entre une tumeur maligne surrénalienne et un adénome [8]. Il est reconnu dans la littérature que les énormes lésions ont une plus grande probabilité d′être maligne. En particulier, les lésions de plus de 4 cm de diamètre ont tendance à être soit une métastase soit un carcinome primitif surrénalien. Le changement de la taille des lésions est un indicateur utile de la malignité, car les adénomes se développent lentement et ont tendance à ne pas changer de taille [7]. La forme de la glande surrénale peut aussi être utile pour prédire la malignité. En effet, les adénomes ont tendance à avoir des marges lisses et une densité homogène, alors que les métastases ou le carcinome primitif surrénalien peuvent être hétérogènes et ont une forme irrégulière. Dans nos deux cas, ces caractères étaient conformes aux descriptions d'autant plus que dans la première observation un bourgeon tumoral est décrit dans la veine cave inférieure sus rénale. Cependant, bien que ces signes soient nécessaires pour différencier une tumeur bénigne d′une masse maligne de la surrénale, ils ne sont pas spécifiques.

Actuellement, il est décrit dans la littérature deux critères majeurs à la TDM basés sur l'histologie et la physiologie qui permettent une approche entre les adénomes bénins des masses surrénaliennes malignes. Il s'agit du contenu lipidique de la masse qui représente la différence anatomique entre les adénomes et les masses malignes et la densité qui représente la différence physiologique. En effet, les adénomes sont riches en matières grasses intracytoplasmiques et ont donc une faible densité au scanner. Si la densité est inférieure à dix unités Hounsfield (UH) sur une acquisition réalisée avant injection de contraste, on peut considérer la masse comme un adénome bénin. La mise en évidence de la graisse dans la lésion est en général suffisante pour arrêter les investigations [2, 9]. A l′inverse, les masses surrénaliennes malignes ou les métastases ont peu de graisses intracytoplasmiques et par conséquent ont une densité élevée sans injection de produit de contraste au scanner. Ces faits sont corroborés dans une étude quantifiant les matières grasses des glandes surrénales. Elle avait montré l'existence d'une forte corrélation entre la teneur riche en matières grasses des glandes surrénales et une faible atténuation à la TDM avec la perte de signal au déplacement chimique à l'imagerie par résonance magnétique [10].

La première étude qui a utilisé la série sans injection au scanner pour caractériser les masses surrénales étiquetées bénignes ou malignes a été réalisée par Lee et al [11]. Dans cette étude, la densité moyenne des adénomes était de 2,2 HU et celle de la tumeur maligne était de 29 HU. Ceci est conforme aux résultats notés chez nos deux patients. Elle était respectivement de 43 UH et de 36 UH dans la seconde. À un seuil de 0 HU, la sensibilité de la TDM sans injection pour la caractérisation des adénomes surrénaliens était de 47%, avec une spécificité de 100%. A un seuil de 10 HU, la sensibilité a changé à 79%, avec une spécificité de 96%. Ces faits ont été confirmés par des études ultérieures [9, 10, 12]. Les seuils pour différencier les lésions bénignes des malignes varient de 0 à 18 HU. En effet, la méta-analyse de Boland et al [12] pour déterminer un seuil optimal de différenciation des lésions bénignes des malignes a noté que la caractérisation des tumeurs surrénaliennes était obtenue au seuil de 10 HU avec une sensibilité de 71% et une spécificité de 98%. Cette spécificité est proche de 100% lorsque d′autres caractéristiques telles que la taille des surrénales, la forme, et les modifications de taille des tumeurs sont pris en compte. La faible atténuation est utilisée pour caractériser un adénome, cependant au moins 30% des adénomes ne contiennent pas suffisamment de lipides, pour avoir une faible atténuation au scanner car elles représentent une population hétérogène [13, 14]. Si bien qu'il est nécessaire d'utiliser une autre méthode diagnostique basée sur la cinétique de rehaussement du nodule après injection de produit de contraste. En effet, si les adénomes et les tumeurs malignes ont chacun un rehaussement important à la phase précoce, à dix minutes, la densité de l'adénome aura diminué de 50% au moins, contrairement à celle de la tumeur maligne qui aura diminué en moyenne d'environ 30% [15]. Cette différence de lavage du produit de contraste a été exploitée afin de mieux différencier les lésions bénignes des lésions malignes surrénales. Dans nos deux cas, Le wash out absolu est de 27% et le relatif de 9% dans la première observation, tandis que dans la deuxième observation, on notait un wash out absolu de 16% et le relatif de 4% faisant évoquer des tumeurs malignes, conformes à la description de Caoili et al. avec une spécificité de 100% [13].

Toutefois, les valeurs d′atténuation des adénomes et des tumeurs malignes peuvent être proches ne permettant pas ainsi de les différencier [10, 15]. Des calcifications surrénaliennes sont facilement mises en évidence en tomodensitométrie. Les carcinomes surrénaliens sont généralement volumineux (40-100 mm) lors de leur découverte. Ils contiennent fréquemment une nécrose centrale et dans 30% des cas des calcifications. Une lésion controlatérale est présente une fois sur dix [10]. Ces faits sont notifiés chez nos deux patients confortant l'hypothèse de tumeurs malignes.

Il est important de souligner que si une lésion ne peut pas être définitivement étiquetée comme adénomateuse après examen TDM, une évaluation plus poussée par l′IRM, la tomographie par émission de protons ou une biopsie de la surrénale doivent être effectuées afin de confirmer la nature bénigne ou maligne des lésions surrénaliennes [16].

## Conclusion

Un examen tomodensitométrique en coupes fines avant et après injection de produit de contraste, avec une séquence tardive à dix minutes est la méthode de choix pour la détection et la caractérisation des anomalies de la glande surrénale. Elle s'impose dans la recherche d’étiologie des tumeurs surrénaliennes. Toutefois, elle devrait être précédée d'examens de dépistage biochimiques appropriés pour différencier un adénome bénin d'une tumeur maligne. Une séquence sans injection de produit de contraste doit être effectuée avec une évaluation de la densité. Toutefois, pour toute masse qui reste indéterminée, l'IRM des glandes surrénales ainsi que le biopsie doivent être réalisées. Enfin, certaines fonctionnalités au scanner peuvent être utilisées par le radiologue pour établir un diagnostic définitif d'une masse surrénalienne basé sur les caractères de la masse surrénalienne.
